# Computed Tomography Findings of Cerebral Venous Thrombosis: A Case Report

**DOI:** 10.5070/M5.52331

**Published:** 2026-07-31

**Authors:** Alex Y Koo, Samuel Shulimson, Joelle Borhart

**Affiliations:** * Georgetown University Medical Center, Department of Emergency Medicine, Washington, DC; ^ MedStar Washington Hospital Center, Department of Emergency Medicine, Washington, DC

## Abstract

Cerebral venous thrombosis (CVT) is an uncommon occurrence, but it carries significant morbidity.^1^ CVT symptoms overlap with other common emergency department conditions, and it requires a specific neuroimaging for accurate detection, making this diagnosis a significant challenge for emergency clinicians. We present a case of a 20-year-old postpartum woman with a history of a post-dural puncture headache following a spinal block with worsening headache and disequilibrium. She was found to have a cerebral venous thrombosis on non-contrast head CT and CT venogram of the head. Her course was uncomplicated with appropriate symptom control while on anticoagulation. We review her CT imaging findings and provide a brief review of the current recommendations and literature on CVT.

**Topics:**

Cerebral venous thrombosis, empty delta sign, CT venogram, headache, dural sinus thrombosis.

**Figure:**
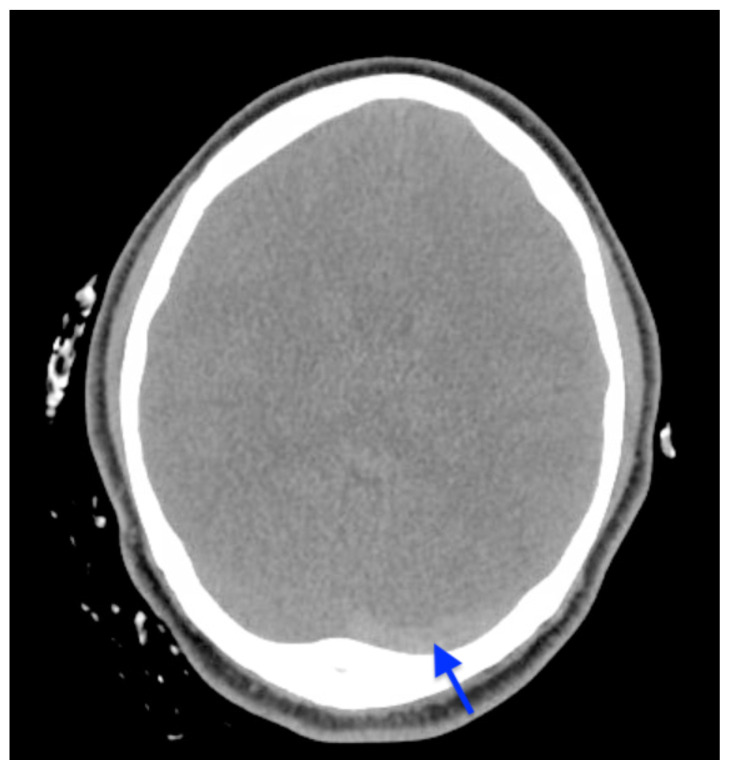


**Figure:**
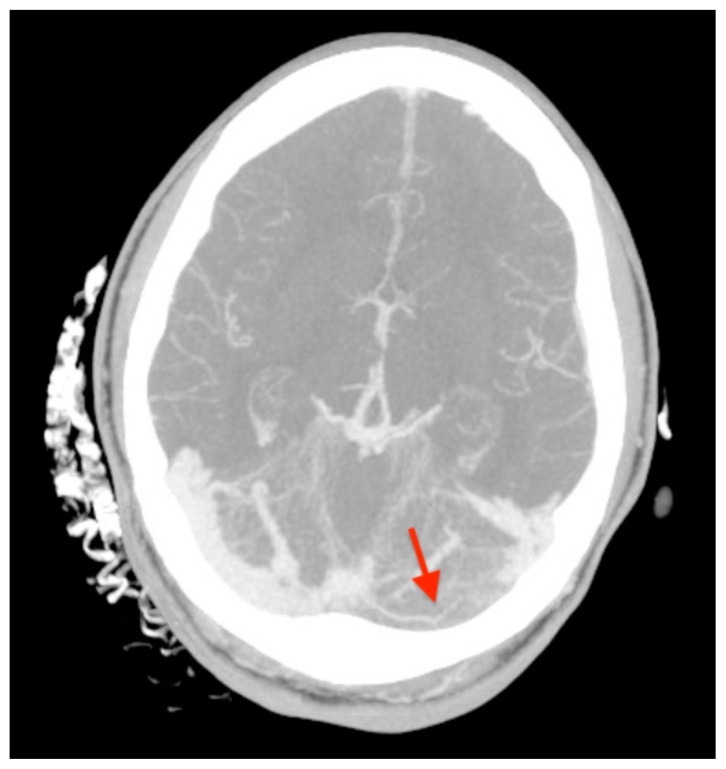


**Figure:**
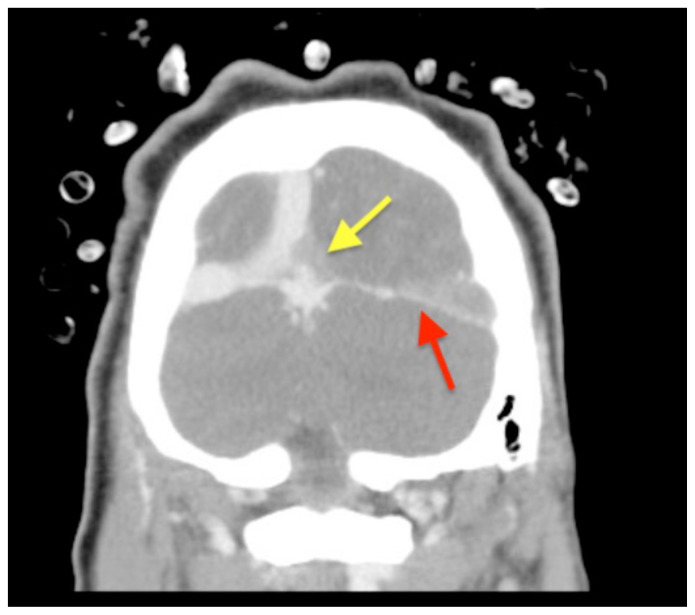


## Brief introduction

Cerebral venous thrombosis (CVT) is a category of strokes, occurring when there is occlusive thrombosis in the dural sinuses and/or cortical veins of the brain.^1^ The smaller cortical veins drain into dural venous channels. The largest dural channel is the superior sagittal sinus, which drains into the transverse sinuses on either side.^1^ The incidence of CVT is rare, accounting for as few as 0.5–3% of all strokes worldwide.^1^,^2^ Risk factors for the development of CVT include inherited or acquired hypercoagulability (eg, peripartum, oral contraceptives), infections, surgery, younger female (<50 years old), obesity, and certain medications.^2^–^4^ Up to 59% of CVT cases occur in the peripartum period, which is the second most common risk factor.^2^ Computed tomography venography (CT-V) or magnetic resonance venogram (MRV) are the best imaging modalities to evaluate for CVT. We describe a case of a 20-year-old postpartum woman presenting with headache, photophobia, and disequilibrium after a spinal block during delivery. She had a non-contrast head CT and CT venogram of the head showing a CVT and had an uncomplicated course after anticoagulation and symptom management.

## Presenting concerns and clinical findings

A 20-year-old gravida 2 para 1 woman who was seven days postpartum following a spontaneous vaginal delivery presented to the emergency department (ED) with headache and photophobia. The patient had a spinal block and then developed a consistent headache, diagnosed as a post-dural puncture heartache, and discharged home. Her recent delivery was complicated by preeclampsia without severe features but was otherwise unremarkable. The patient had been managing her post-dural puncture headache with ibuprofen and acetaminophen with adequate pain control. However, she experienced worsening headache, photophobia, and disequilibrium prompting her visit to the emergency department. She had to be assisted by her partner to walk into the ED from the car.

The patient’s initial vital signs were normal. She was alert, in a dark room, and wearing sunglasses. There were no focal neurological deficits, no truncal ataxia, and finger-to-nose testing was normal. However, she experienced significant occipital head pain and nausea with sitting up. The epidural site was clean, dry, and intact. The rest of her exam was normal with decreasing lochia, no fevers or abdominal pain.

## Significant findings

The emergency physician ordered a non-contrast head CT and CT venogram of the head given worsening symptoms with disequilibrium and CVT risk factors of postpartum coagulopathy. The non-contrast head CT demonstrated a hyperdensity occlusion of the left transverse sinus (blue arrow). This serpiginous hyperdensity is called the “cord” or “string” sign, which is thrombosis within the vein.^2^ The CT venogram of the head demonstrated occlusion of the left transverse (red arrows) and sigmoid sinus (yellow arrow).

## Patient course

The ED physician ordered metoclopramide, ketorolac, and acetaminophen with improvement of her headache and nausea. Subsequently, the ED physician started the patient on heparin and consulted neurosurgery, neurology, and obstetrics. No acute interventions were needed from the neurosurgical team, and the neurology and obstetrics teams recommended admission to the step-down unit for frequent neurological checks. The patient was transitioned to low-molecular-weight-heparin (LMWH) given its efficacy for CVT and compatibility with breastfeeding. Her inpatient course was uncomplicated, and the patient was discharged on hospital day #1 with LMWH for 3 months, a repeat CT venogram of the head, and follow-up with neurology prior to discontinuing anticoagulation.

## Discussion

Headaches occur nearly in 90% of patient with CVT. However, headache is a common presenting symptom in the ED with many etiologies which make the diagnosis of CVT difficult. Other signs and symptoms related to increased intracranial pressure are vision loss (13–27%) seizures (20–40%), and focal neurological deficits (20–50%).^1^,^2^,^5^ Furthermore, non-contrast head CT alone is not particularly sensitive for the diagnosis of CVT, with 41–73% sensitivity and 97–100% specificity. CT venogram of the head approaches 95% sensitivity and specificity for diagnosing CVT, compared to MRV.^3^,^6^,^7^ Clinicians must have a high suspicion for CVT to order the recommended imaging (i.e., CT-V or MRV).

Our case demonstrates the non-classical presentation of CVT. While the patient presented with headache (common for CVT), she did not present with findings of severe increased intracranial pressure such as seizures or vision loss. She did have positional variation with her headaches, which could be a sign of CVT, but also overlaps with a post-dural puncture headache. Patients with CVT may present with focal neurological deficits or seizures in up to 50% of cases, which our patient did not have.^2^ In this case, the treating clinician had increased suspicion for CVT given the acute change to the quality of her headache as well as being seven days postpartum with disequilibrium. The strongest risk factors for CVT are being within six weeks postpartum and use of oral contraceptives, each increasing the risk approximately eightfold.^2^

Our patient’s CT-V demonstrated a left transverse and left sigmoid thrombosis. Traditionally, the “empty delta sign” on CT-V or “dense triangle sign” on non-contrast head CT may be seen. These findings are the pathognomonic finding of thrombosis in the superior sagittal sinus, which is the most common CVT location. In our case, there was no “empty delta sign” or “dense triangle sign” since the patient had a patent superior sagittal sinus. Instead, a filing defect was noted in the left transverse and sigmoid sinuses on CT-V.^3^

The 2024 American Heart Association/American Stroke Association recommends anticoagulation with low-molecular-weight heparin (LMWH) with transition to vitamin K antagonists for 3–12 months in the context of transient risk factors. In cases with significant mass effect or herniation, neurosurgical intervention (eg, decompressive hemicraniectomy) may be indicated. Other neurosurgical interventions such as endovascular thrombectomy or thrombolysis can be considered if there is clinical or imaging progression.^2^,^6^,^8^

This case emphasizes the importance of maintaining a high index of suspicion for CVT in postpartum patients and the recognition of the superiority of CT venogram of the head for diagnosing CVT. Appropriate anticoagulation for CVT includes heparin and LMWH, since these are both safe during pregnancy and breastfeeding. Finally, it is important to consider the increased risk of venous thromboembolism with future pregnancies.^2^

## Supplementary Information












